# Psychometric validation of an empowerment scale for Spanish-speaking patients with rheumatoid arthritis

**DOI:** 10.1186/s13075-018-1741-6

**Published:** 2018-10-30

**Authors:** Irazú Contreras-Yáñez, Emmanuel Ruiz-Medrano, Luz del Carmen R. Hernández, Virginia Pascual-Ramos

**Affiliations:** 1 Department of Immunology and Rheumatology, Instituto Nacional de Ciencias Médicas y Nutrición Salvador Zubirán, Vasco de Quiroga 15, Colonia Belisario Domínguez Sección XVI, México City, Mexico; 2 External collaborator, Mexico City, Mexico

**Keywords:** Rheumatoid arthritis, Empowerment scale, Psychometric validation

## Abstract

**Background:**

Rheumatoid arthritis (RA) knowledge has been constructed with studies performed in Caucasians patients; Latin American patients present unique characteristics. Empowerment is a social multidimensional construct that has been associated to better health-related quality of life in RA. There is no validated instrument for use with Spanish-speaking patients. The objective of the study was to adapt the Spanish version of the Health Empowerment Scale (S-HES), which was selected for its psychometric properties and suitability for low-literacy populations, for RA Hispanic patients (RAEH), and to perform its psychometric validation.

**Methods:**

RAEH adaptation, pilot testing, and psychometric validation were performed. Three convenience samples of RA outpatients from a national tertiary care level center were used. For RAEH adaptation, the word “health” was substituted with “RA” in the original S-HES, integrated by 8 items. Pilot testing (in 50 patients) assessed feasibility. Psychometric validation included content validity (nine experts rated item convenience, clarity, and cultural semantic accuracy), internal consistency (in 200 patients, Cronbach’s alpha) and test–retest (in a subsample of 50 patients, ICC and 95% CI), construct validity (factor analysis), and face validity (in 20 patients, % of agreement). Patients gave written informed consent.

**Results:**

Patients were primarily middle-aged females and had typical long-standing disease, although early disease was represented. In the psychometric validation sample, the majority of the outpatients had autoantibodies; meanwhile, half of them had no evidence of disease activity, with acute reactants phase determinations within normal range. Patients with comorbidities and joint replacement were also included.

Experts agreed upon the attributes of content validity: 83–100% considered the item was essential, 100% agreed on the item’s clarity and 80–100% on the cultural semantic accuracy. In the pilot sample, ≥ 80% of the patients agreed with the item’s clarity and format.

In the psychometric validation sample, mean RAEH was 34 (maximum possible score: 40 = highest score). RAEH had a good internal consistency, Cronbach’s α = 0.86, and moderately good reliability (ICC [95% CI] test–retest: 0.79 [0.62–0.88]). Factor analysis for construct validity showed a single factor explaining 52% of the variance.

Patients agreed with each item content validity (85–100%) and clarity (75–100%).

**Conclusions:**

RAEH was valid and reliable to evaluate empowerment in Spanish-speaking RA patients.

**Electronic supplementary material:**

The online version of this article (10.1186/s13075-018-1741-6) contains supplementary material, which is available to authorized users.

## Background

Patient empowerment is a social multidimensional construct [1], largely accomplished by individuals themselves. Most empowerment definitions focus on the individual’s capacity to make decisions about their health-related behavior and to have or take control over aspects of their lives that relate to health [2, 3]. Empowerment has been conceived as a health-enhancing process [3, 4], as a measurable health-related psychosocial outcome [2, 5] and even as an aspect of health-related quality of life (HRQoL) [6]. Currently, there is not a universal definition of empowerment and it has been argued that the construct may be context- and population-specific [7]; culture, age and socioeconomic resources influence empowerment level and the degree to which the different social groups can be and wish to be empowered differ [2]. A study performed in 33 developing countries, 21 of them African, demonstrated considerable variation between countries with regard to the relationship between women’s empowerment and their use of maternal health services; the authors highlighted the need to develop locally sensitive and meaningful measures of (women’s) empowerment [8]. In the field of chronic (non-rheumatic) diseases, empowerment facilitates patients’ involvement in their care and has been related to treatment safety and effectiveness [9, 10], to lower dependence on health services [9, 11], to better treatment adherence, and to improvement in specific health outcomes [9, 10].

Rheumatoid arthritis (RA) is a worldwide chronic inflammatory disease that impacts patients’ HRQoL and may result in increased mortality. Early and aggressive treatment with disease modifying anti-rheumatic drugs (DMARDs) improves outcomes [12]. The 2013 European League against Rheumatism (EULAR) treatment-recommendations for RA emphasizes that treatment must be based on a shared decision between the patient and the rheumatologist [12]. In order to have patients form a partnership with their primary physician in optimizing their health-related disease, patients require knowledge about their disease, skills to self-manage their disease and participate in medical decision making, and power [13]; all of them reflect empowerment attributes.

RA patients from Latin American countries present distinctive characteristics; the literature highlights a lower prevalence [14], a younger age at presentation [14, 15], and a higher female:male ratio [14, 15] in this population, compared with Caucasians. In addition, patients from Latin America present individual and situational sources of vulnerability [14–16] that affect compliance with medications and negatively impact outcomes [15, 17]. Two studies assessed empowerment in RA patients and its association with patient-related outcomes. In the first one, performed in the USA, RA patients found an association between enhancing patients’ empowerment (no validated scale was used) and improvement in some 36-item Short Form (SF-36) categories [18]. The second one was performed in Swedish patients with rheumatic diseases (including RA) and showed an association between patients’ empowerment, as assessed according to a scale that was validated in the same study, and better self-reported health [19]. Few additional studies described empowerment-enhancing approaches in RA patients, although no validated scales were used [20–23]. None of these studies had been performed in Latin American populations, which limits the comprehensiveness of the topic.

Assessment of empowerment requires the use of validated tools with appropriate psychometric properties. A recent systematic review detailed 19 measures with varying quality, 6 of them generic and 13 developed for a specific condition [24]. In addition, a Spanish questionnaire to assess empowerment for self-care has been described, although limited to climacteric women [25]. The Swedish Rheumatic Disease Empowerment Scale (SWE-RES-23) is the only questionnaire adapted for rheumatic diseases and was developed in Swedish [19]. The scale, adapted from the Swedish version of a Diabetes Empowerment Scale, showed adequate psychometric properties in Swedish patients with rheumatic diseases; there is no Spanish version. The Spanish version of the Elders Health Empowerment Scale (S-HES) is a generic scale that reflects the attributes of empowerment construct; its psychometric properties were tested in urban senior citizens (men and women) from Rosario, Argentina [26]. The literature highlights important differences between Hispanic and non-Hispanic Whites, particularly in regard to health-related decision making [27], which is conceptually close to empowerment construct. In addition, the S-HES has shown good validity and reliability (in Spanish-speaking people), is easy to apply as the instrument includes 8 items, and is considered simple to understand according to an elementary literacy level [26, 28], which is relevant for Latin American countries. Based on those considerations, we selected the S-HES, adapted the scale as an RA Empowerment Scale for Hispanic patients (RAEH), and perform its psychometric validation. We present herein the results.

## Methods

The study was conducted in three steps: (1) RAEH adaptation, (2) pilot testing, and (3) psychometric validation.

### Description of samples

Three different convenience samples of consecutive RA patients were considered; patients included in each sample belonged to the outpatient clinic of the Instituto Nacional de Ciencias Médicas y Nutrición Salvador-Zubirán (INCMyN-SZ), a tertiary care level and national referral center for rheumatic diseases. All the patients had RA diagnosis, which was established according to their primary physician criteria (rheumatologist or trainee in rheumatology, no validated classification criteria were used), who additionally classified each patient as in remission or with active disease (no standard definition was used). In addition, all the patients had major comorbidity assessed according to the Charlson score [29]. In the three samples, the patients’ selection was directed to have age, gender (female preponderance), disease duration, and disease activity quotes represented.

The first sample included 50 patients and was used to evaluate feasibility; the second one included 200 patients and was used for the psychometric validation; the last sample included 20 patients and was used for face validity.

### Sample size calculation for psychometric validation

Considering the number of RAEH items (8 items), a sample size of at least 80 RA outpatients was required as the exploratory factor analysis recommends 5 to 10 respondents per item [30]. However, taking into account additional published recommendations (at least 150 to 200 patients), we decided to include 200 RA patients (fair sample size) in the final sample [31].

### Step (1) RAEH adaptation (see Additional file 1)

The S-HES author was contacted in order to have him involved in the process. The RAEH was adapted from the S-HES, that retained 8 items (one per subscale), with 8 subscales: satisfaction and dissatisfaction related to health, identification and achievement of personally meaningful goals, application of a systematic problem-solving process, coping with the emotional aspects of living with health, stress management, appropriate social support, self-motivation and making cost/benefit decisions about making behavior changes. Each item is scored on a 5-point Likert scale, ranging from 5 (strongly agree) to 1 (strongly disagree). In the scale, higher scores indicate stronger level of health-related empowerment, with scale scores ranging from 1 to 5.

First, in each item of the S-HES, the word “health” was substituted by “rheumatoid arthritis”. Then, each of three researchers (one rheumatologist, one trainee in rheumatology and one social worker, a PhD candidate in Health Sciences) suggested one sentence per item, in order to have different perspectives represented; researchers were blinded to each other proposals. After a consensus was obtained, the three researchers selected one sentence per item and integrated a preliminary version of the RAEH. RAEH was scored as in the original scale, but the sum of individual item-scores was provided; accordingly, RAEH score ranged from 8 to 40.

### Step (2) Pilot testing

Feasibility was tested (in 50 patients) according to the following criteria: time required to fill the scale, patients’ perceived item’s clarity, and patients’ format acceptance.

### Step (3) Psychometric validation

#### Content validity

Content validity was tested by a Validation Expert Committee (VEC) that was integrated by six rheumatologists and two psychiatrists who received relevant literature related to empowerment construct, and the author of the original S-HES (a geriatrist and psychiatrist). Content validity was examined by asking members of the VEC to rate each of the eight sentences (one per item) according to three categories: unnecessary, important but not necessary or essential. In addition, the VEC rated the item’s clarity and cultural semantic accuracy.

#### Reliability

Internal consistency (assessed in 200 patients) and test–retest (assessed in a subsample of 50 patients, by the same researcher, with an interval of 3 ± 1 weeks) were evaluated to determine the reliability of the scale.

#### Construct validity

Construct validity was determined with factor analysis.

#### Face validity

Previously, a brief explanation of the empowerment concept was offered to 20 patients, who were, in a second step, directed to rate each item as a valid measure of the respective empowerment dimension (Yes/No) along with each sentence’s clarity (Yes/No).

### Statistical analysis

Descriptive statistics was performed to estimate the frequencies and percentages (for categorical variables), means and standard deviation (SD) or medians and 25th–75th interquartile (IQ) ranges (for continuous variables) of sociodemographic- and disease characteristics-related variables.

RAEH construct validity was evaluated by confirmatory factor analysis (maximum likelihood) with Varimax rotation. Sampling adequacy was confirmed by the Kaiser-Mayer-Olkin (KMO) measure (appropriate value ≥ 0.5); use of factor analysis was supported by the Bartlett’s test of sphericity (significant value *p* < 0.05), eigenvalue > 1 and correlations coefficients > 0.30 [32]. Floor and ceiling effects were determined as the percentage of patients who achieved the lowest and highest score of the scale, respectively.

Cronbach’s α and inter-item correlation for the complete scale and for each dimension was used to assess RAEH internal consistency of the questionnaire. Cronbach’s alpha interpretation was as follows: < 0.70 indicates that individual items provide an inadequate contribution to the overall scale and values of > 0.90 suggest redundancy, [33].

RAEH test–retest reliability was evaluated by the *t* test comparison between total test scores and by between partial test dimension score. In addition, intra-class correlation coefficients (ICC) and their 95% confident intervals (CI) were calculated based on a single measurement, absolute-agreement, two-way mixed-effects model. According to the ICC, values < 0.5 indicate poor reliability, between 0.5–0.75 moderate reliability, between 0.75 and 0.9 good reliability and values > 0.9 indicate excellent reliability. Finally, 95% CI estimates between 0.83 and 0.94 were considered as good reliability level and those between 0.95 and 0.99 estimates, as excellent reliability level [34].

All statistical analyses were performed with Statistical Package for the Social Sciences version 21.0 (IBM Corp., Armonk, NY, USA). A value of *p* ≤ 0.05 (two tails) was considered to be statistically significant.

### Ethical considerations

The study received ethical approval from the Comité de Ética en Investigación of the INCMyN-SZ (reference number: 2226-17/ 18-1. Written informed consent was obtained from all the patients who agreed to participate.

## Results

### Description of the population characteristics of the three samples

During the study, there were 270 RA outpatients recruited; they were divided in three samples. Table 1 summarizes their characteristics. Patients were primarily middle-aged females, married, had basic education and medium-low socioeconomic status. The population had long-standing disease although patients with ≤ 5 years of disease duration were also represented.

**Table 1:** Description of the population characteristics of the three samples

Population characteristics	*N* = 50 (Feasibility)	N = 200 (Psychometric validation)	N = 20 (Face validity)
Females, N (%)	46 (92)	184 (92)	19 (95)
Age, years	55 (47–63)	52 (41–61)	52 (32–61)
Marital status, patients married, *N* (%)	27 (54)	113 (56.5)	19 (95)
Formal education, years	9 (9–11)	9 (9–12)	12 (9–17)
Patients with medium-low SE level, *N* (%)	47 (94)	186 (98)	16 (80)
Disease duration, years	12 (6.8–19)	12 (6–18)	11 (3–5)
Patients with disease duration ≤5 years, *N* (%)	10 (5)	44 (22)	7 (35)
Patients with remission, *N* (%)	NA	107 (53.5)	NA
CRP, mg/dL	NA	0.57 (0. 18–1.5)	NA
ESR, mm/H	NA	12 (5–24)	NA
Patients with RF, N (%)	NA	179 (89.5)	NA
Patients with ACCP, N (%)	NA	134 (72.4)*	NA
Patients with major comorbidities, N (%)	11 (22)	38 (19)	4 (10)
Patients with joint replacement, N (%)	5 (10)	30 (15)	2 (10)

Data as presented as median (25th–75th interquartile range) unless otherwise indicated *ACCP* antibodies to cyclic citrullinated peptides, *CRP* C-reactive protein, *ESR* erythrocyte sedimentation rate, *N* number, *NA* not available, *SE* socioeconomic, *RF* rheumatoid factor. *Data limited to 185 patients with sample available

In the sample for the psychometric validation, more than half of the patients were in remission and had acute reactants phase determinations (erythrocyte sedimentation rate [ESR] and C-reactive protein [CRP]) within normal range; also, the majority of the patients had disease-specific autoantibodies, particularly rheumatoid factor (RF) and antibodies to cyclic citrullinated peptides (ACCP).

Finally, in the three samples, patients with a major comorbid condition and patients with a surgical joint replacement were also represented.

Patients and disease characteristics were similar in the three samples, but patients from the face validation sample were more frequently married and had more disease duration ≤ 5 years (*p* = 0.02 and *p* = 0.03, respectively).

### Feasibility

Early during pilot testing (five patients enrolled), patients manifested a preference for a different order in the item’s disposition; the suggestion was adopted and maintained in the final version of the RAEH used during the validation process (see Additional file 2).

(Mean) time required to fill the scale was 7 min and all the patients agreed the time was convenient. Eighty-five percent of the patients agreed on the item’s clarity and 95% of them agreed on the scale format.

### Content validity

The preliminary version of the RAEH was integrated by 8 items, one per subscale. This version was submitted the VEC who agreed that each sentence/item was essential (83–100%), and about each item clarity (100%) and cultural semantic accuracy (100%), as summarized in Table 2.

**Table 2:** RAEH content validity according to the Validation Expert Committee (VEC)

Empowerment subscales	Items	VEC*		
		Essential	Clarity	CS accuracy
Self-control	I know what parts of taking care of my rheumatoid arthritis that I am dissatisfied with.	83	100	100
Self-efficacy	I am able to achieve my rheumatoid arthritis goals through concrete actions.	100	100	100
Problem solving	I can try out different ways of overcoming barriers to achieve my rheumatoid arthritis goals.	83	100	100
Psychosocial coping/Coping with emotional aspects	I can find ways to feel good having rheumatoid arthritis.	100	100	100
Psychosocial coping/Stress management	I can use positive ways to cope with rheumatoid arthritis-related stress.	100	100	80
Support	I can find support to care for my rheumatoid arthritis.	83	100	100
Motivation	I recognize what helps me stay motivated to care for my rheumatoid arthritis.	83	100	100
Decision making	I know enough about myself to make rheumatoid arthritis care choices that are the most convenient to me.	83	100	100

*CS* cultural semantic (accuracy), *RAEH* Rheumatoid Arthritis Empowerment Scale for Hispanic patients

*% of agreement among members of the VEC

### Reliability

The median RAEH score for the sample was 34 (31–37), with a minimum possible score of 8 and a maximum possible score of 40. Coefficient of Kurtosis (4.5) and skewness (1.29) showed non-normal data distribution. RAEH exhibited good internal consistency, with Cronbach’s α = 0.86 for the full scale. Floor and ceiling effects were of 0.5% and 7.5%, respectively; for individual items, the percentage of patients scoring at the floor was 0.5–2% and the percentage of patients scoring at the ceiling was 26. 5–52.5%. Details of statistics, Cronbach’s α, inter-item correlation and floor and ceiling effects for each particular item are summarized in Table 3.

**Table 3:** Statistics, corrected item-total correlation, floor and ceiling effects, and factorial loading for RAEH items (N = 200)

Item	Median (25th–75th IQ) score	Corrected item-total correlation	Floor effect (% scoring 1)	Ceiling effect (% scoring 5)	α if item delete	Factorial loading
1	4 (4–5)	0.544	0.5	26.5	0.854	0.594
2	4 (4–5)	0.566	1	41.5	0.851	0.612
3	4 (4–5)	0.636	0.5	40	0.844	0.674
4	5 (4–5)	0.671	0.5	52.5	0.840	0.719
5	4 (3–5)	0.508	1.5	27	0.860	0.566
6	4 (4–5)	0.670	0.5	38.5	0.839	0.729
7	4 (4–5)	0.666	2	40	0.839	0.735
8	4 (4–5)	0.673	0.5	42.5	0.839	0.735

Item1 = I can use positive ways to cope with rheumatoid arthritis-related stress

Item 2 = I can find support to care for my rheumatoid arthritis

Item 3 = I recognize what helps me stay motivated to care for my rheumatoid arthritis

Item 4 = I know enough about myself to make rheumatoid arthritis care choices that are the most convenient to me

Item 5 = I know what parts of taking care of my rheumatoid arthritis that I am dissatisfied with

Item 6 = I am able to achieve my rheumatoid arthritis goals through concrete actions

Item 7 = I can try out different ways of overcoming barriers to achieve my rheumatoid arthritis goals

Item 8 = I can find ways to feel good having rheumatoid arthritis

*IQ* interquartile, *RAEH* Rheumatoid Arthritis Empowerment Scale for Hispanic patients

Mean (±SD) time between the two measurements in the test–retest analysis was of 19.3 (±6.9) days; ICC was of 0.79 (95% CI: 0.62–0.88, *p* ≤ 0.001). Finally, comparison of means of the total score and each dimension-score between the two measurements did not significantly differed (data not shown).

### Construct validity

Construct validity was demonstrated by KMO = 0.884 and Barlett’s test of sphericity (X^2^ = 610.93, *p* ≤ 0.001) which confirmed the adequacy of the sample size for conducting factor analysis. A single factor structure was extracted, accounting for 52% of the variance. Table 3 summarizes results for items construct validity.

### Patients’ RAEH face validity

Table 4 summarizes results from this process; the majority of the patients agreed that the items were a valid measure of the corresponding dimension (85–100%) and that items were clear (75–100%).

**Table 4:** Patients’ RAEH face validity (*N* = 20)

Items	Valid measure*	Clarity/readability*
I can use positive ways to cope with rheumatoid arthritis-related stress.	19 (95)	19 (95)
I can find support to care for my rheumatoid arthritis.	20 (100)	20 (100)
I recognize what helps me stay motivated to care for my rheumatoid arthritis.	20 (100)	19 (95)
I know enough about myself to make rheumatoid arthritis care choices that are the most convenient to me.	19 (95)	20 (100)
I know what parts of taking care of my rheumatoid arthritis that I am dissatisfied with.	17 (85)	15 (75)
I am able to achieve my rheumatoid arthritis goals through concrete actions.	18 (90)	19 (95)
I can try out different ways of overcoming barriers to achieve my rheumatoid arthritis goals.	20 (100)	20 (100)
I can find ways to feel good having rheumatoid arthritis.	20 (100)	20 (100)

*RAEH* Rheumatoid Arthritis Empowerment Scale for Hispanic patients

*N (%) of patients with agreement

### Handling of incomplete questionnaires

Only 10 scales (5% of the psychometric validation sample) had ≤ 1 missing response/item. There was no consistency in the items with omitted response. In all cases, patients stated that they skipped it by mistake. No item was considered objectionable or did not apply to the patient. Incomplete scales were returned back to the patients, who filled them in.

## Discussion

In the present study, we performed a psychometric validation of the RAEH, which was adapted from a generic scale that assessed health-related empowerment. RAEH psychometric properties were adequate in terms of construct, content and face validity, and reliability, which were evaluated with internal consistency and test–retest, as recommended [30]. In addition, RAEH was also feasible based on users’ evaluation. Importantly, different samples of consecutive RA outpatients were used to perform analysis. We suggest that RAEH can be used to evaluate empowerment in Hispanic RA patients; the scale is simple and suitable for low-literacy patients and its use could be generalized to Spanish-speaking RA patients from other countries. Although ethnic heterogeneity is characteristic of the Latin American population, RA patients from this geographic area present distinctive characteristics when compared to white populations from the United States and Europe [35].

The RAEH showed adequate internal consistency reliability; Cronbach’s α coefficient for the total scale and the eight empowerment dimensions were good, with α value of 0.86 [33]. The test–retest reliability assessed in 50 patients by the same researcher showed an ICC of 0.79, indicating good reliability [34], with 95% CI of 0.62–0.88. The construct validity was demonstrated by KMO sampling and Barlett’s test of sphericity, both confirming the adequacy of the sample size for conducting factor analysis [33], and a single factor structure was extracted, accounting for 52% of the variance. Face and content validity were examined by patients, and a multidisciplinary committee of health care providers involved in RA management. Patients (RAEH face validity) agreed that items were a valid measure of the corresponding dimension and that items were clear; additional patients confirmed RAEH feasibility. Patients involvement may be particularly relevant as one would expect the indicators of empowerment to be defined by the patients themselves, rather than (or in addition to) by health care professionals. Finally, the RAEH (total score) did not show neither floor nor ceiling effect, which had been defined when more than 15% of the patients achieved the lowest or highest score, respectively [30]. Both, floor and ceiling effects can reduce the possibility of detecting change over time.

This is the first scale validated in the Hispanic population that measures empowerment in patients with RA. There is only one additional scale, the SWE-RES-23, which assessed empowerment in patients with rheumatic diseases [19]. The scale was developed in Swedish and psychometric properties were evaluated in a population primarily represented by middle-aged females; in addition, although patients with different rheumatic diagnosis were included, 74% of them were classified with inflammatory joint diseases, which include RA diagnosis. The RAEH and the SWE-RES-23 showed similar psychometric properties in terms of construct validity, internal consistency and reliability although stability reliability was not tested in the SWE-RES-23, meanwhile the RAEH showed good ICC. Regarding RAEH ceiling effects, it should be mentioned there were ceiling effects in all the individual’s dimensions, which indicates that patients start with higher empowerment skills than the average and may lack room for improvement [30]. Arvidsson et al. also found ceiling effects in two subscales of the SWE-RES-23 and the total score [19], as did Serrani in six out of eight subscales of the S-HES [25] (see Additional file 3).

The median RAEH score for our target population was 34, which confirms that our patients showed an empowerment level above medium values, as has been found in other studies [19, 25]. The average of the empowerment measurement cannot be compared between the RAEH and the SEW-RES-23, because they had different number of reagents and dimensions. Nonetheless, total RAEH mean value (4.2 ± 0.57) was superior to that from either the SWE-RES-23 (vs. 3.53 ± 0.57, *p* = 0.001) or the S-HES (vs. 3.51 ± 0.73, *p* = 0.001). Our higher scale mean value may be considered unexpectedly high for a population with limited literacy, but could be explained by the long-term RA duration present in our patients (12 years of disease duration).

Additional studies have tried to measure the level of empowerment in patients with RA; however, empowerment has been measured in a subjective manner. Geller et al. [18] used an empowerment program for 6 months in patients with chronic pain (six of them had RA) and assessed the quality of life, but did not quantitatively measure the level of empowerment. Allam et al. [36] tested the effects of online social support and gamification on health outcomes in 157 RA patients; empowerment levels changed over time, more so in the groups having access to online support or a gamified experience of the website. van der Vaart et al. [37] applied questionnaires and measured use, satisfaction and impact of a web portal, which provided patients with home access to their electronic medical records, in 360 RA patients; only 54% of the respondents (to the questionnaires) had viewed their electronic medical records; among those who had logged in, 44% reported feeling more involved in their treatment, 37% felt they had more knowledge about their treatment, but differences over time were not found on the empowerment-related instruments. Different empowerment-enhancing approaches had been described in RA patients in order to support the patients [20] and promote patient independence [21]. Clinical trials may be considered complex systems, and patients enrolled explained their participation as an experience that substantially improved their control and adaptation by a better understanding of their disease, which may ultimately enhance empowerment [22]. Similarly, alternative models of RA patients’ follow-up, whereby patients initiate appointments themselves, may offer greater self-management of the underlying rheumatic disease [23].

The study has some limitations. First, we did not assess RAEH response to change, neither had indication of minimal important change nor minimal important difference, and without such information, it is impossible to understand if changes in levels of empowerment matters to patients [24]. Second, the RAEH was adapted and validated in a particular population of Hispanic RA outpatients from México and, given cultural differences in Latin America, results present here need to be reproduced in other Spanish-speaking countries. Nonetheless, the RAEH is simple and easy to apply particularly in populations with poor levels of literacy. Third, the RAEH questionnaire assessed empowerment skills at the individual level, but did not account for organizational and community levels that may additionally impact empowerment, particularly in patients with chronic conditions [7]. Finally, the RAEH evaluates empowerment limited to RA; nonetheless, empowerment is a complex construct and it may be necessary to set up a disease-specific context to understand empowerment implications, in terms of outcomes relevant to patients.

## Conclusions

In conclusion, RAEH is valid, reliable, and feasible to evaluate empowerment in Spanish-speaking RA patients. Patients from Latin America present unique characteristics and research needs to focus on such populations in order to complete the picture of RA, knowledge of which has been constructed based on studies performed primarily in Caucasians patients. There is (qualitative) evidence in non-rheumatic diseases suggesting that enhancing patient empowerment over their health is highly valued and enables patients to better manage their lives. These, if reproduced in patients with rheumatic diseases, in particular RA, should be conceived as a valuable outcome. The RAEH could be applied in a practical way in studies that use empowerment-enhancing programs in Spanish-speaking RA patients.

## Additional files

Additional file 1: **Table S1.** Items head-to-head comparison between the S-HES and the RAEH. Table shows the items in Spanish and English of the S-HES and RAEH scales. (PDF 304 kb)

Additional file 2: **Figure S1.** Rheumatoid Arthritis Empowerment Scale for Hispanic patients (RAEH). Final version of the Spanish version of the Health Empowerment Scale for RA Hispanic patients used during the validation process. (PDF 568 kb)

Additional file 3: **Table S2.** Comparison of SWE-RES-23, S-HES and RAEH psychometric validation properties. Table shows the statistics of the tests carried out in the validation of the SWE-RES-23, S-HES, and RAEH scales. (PDF 301 kb)

## Abbreviations

ACCP: Antibodies to cyclic citrullinated peptide
C-RP: C-reactive protein
ESR: Erythrocyte sedimentation rate
HRQoL: Health-related quality of life
ICC: Intra-class correlation coefficient
INCMyN-SZ: Instituto Nacional de Ciencias Médicas y Nutrición Salvador Zubirán
KMO: Kaiser-Meyer-Olkin sampling
RA: Rheumatoid arthritis
RAEH: Rheumatoid Arthritis Empowerment Scale for Hispanic patients
RF: Rheumatoid factor
SD: Standard deviation
S-HES: Spanish-Health Empowerment Scale
SWE-RES-23: Swedish Rheumatic Disease Empowerment Scale
VEC: Validation Expert Committee
