# Supplementary material for: Psychometric validation of an empowerment scale for Spanish-speaking patients with rheumatoid arthritis
Source: Arthritis Res Ther. 2018 Oct 30;20:244. doi: 10.1186/s13075-018-1741-6 (PMC6235224; doi:10.1186/s13075-018-1741-6)
Supplement: Supplementary file 1 — Table S1. Items head-to-head comparison between the S-HES and the RAEH. Table shows the items in Spanish and English of the S-HES and RAEH scales. (PDF 304 kb) [file 13075_2018_1741_MOESM1_ESM.pdf]

Table S1. Items head-to-head comparison between the S-HES and the RAEH.

Sub-scales	S-HES<br><br>Spanish/English item (1/subscale)	RAEH<br><br>Spanish/English item (1/subscale)
Self-Control	Sé muy bien con que parte(s) del cuidado de mi salud no estoy satisfecho	Conozco bien con qué aspectos del cuidado de mi artritis reumatoide estoy insatisfecho
	I know what part(s) of taking care of my health that I am dissatisfied with.	I know what parts of taking care of my rheumatoid arthritis that I am dissatisfied with.
Self-efficacy	Soy capaz de alcanzar mis metas de salud mediante planes concretos de acción	Soy capaz de alcanzar las metas que me he propuesto para mi artritis reumatoide mediante acciones concretas
	I am able to turn my health goals into a workable plan.	I am able to achieve my rheumatoid arthritis goals through concrete actions
Problem solving	Tengo diferentes maneras de superar los obstáculos para lograr mis objetivos de salud	Puedo hacer diferentes cosas para superar los obstáculos y lograr las metas que me he propuesto para mi artritis reumatoide
	I can try out different ways of overcoming barriers to my health goals.	I can try out different ways of overcoming barriers to achieve my rheumatoid arthritis goals.
Psychosocial coping/Cooping with emotional aspects	Tener salud me hace sentir mejor	Puedo encontrar maneras para sentirme bien teniendo artritis reumatoide
	I can find ways to feel better about having health.	I can find ways to feel good having rheumatoid arthritis.
Psychosocial coping/Stress management	Puedo afrontar el estrés por mis problemas de salud de manera positiva	Puedo afrontar de manera positiva el estrés que me causa la artritis reumatoide
	I Know the positive ways I cope with health-related stress.	I can use positive ways to cope with rheumatoid arthritis-related stress.
Support	Puedo solicitar ayuda para cuidar y mantener mi salud cuando lo necesito	Puedo encontrar apoyo para cuidar de mi artritis reumatoide
	I can ask for support for having and caring for my health when I need it.	I can find support to care for my rheumatoid arthritis.

Motivation	Reconozco lo que me motiva para cuidar mi salud	Reconozco lo que me ayuda para estar motivado para cuidar de mi artritis reumatoide
	I know what helps me stay motivated to care for my health	I recognize what helps me stay motivated to care for my rheumatoid arthritis.
Decision making	Me conozco lo suficiente para escoger lo que más conviene a mi salud	Me conozco lo suficiente para elegir lo que más me conviene para el cuidado de mi artritis reumatoide
	I know enough about myself as a person to make health care choices that are right for me.	I know enough about myself to make rheumatoid arthritis care choices that are the most convenient to me.
