# Supplementary material for: Psychometric validation of an empowerment scale for Spanish-speaking patients with rheumatoid arthritis
Source: Arthritis Res Ther. 2018 Oct 30;20:244. doi: 10.1186/s13075-018-1741-6 (PMC6235224; doi:10.1186/s13075-018-1741-6)
Supplement: Supplementary file 2 — Figure S1. Rheumatoid Arthritis Empowerment Scale for Hispanic patients (RAEH). Final version of the Spanish version of the Health Empowerment Scale for RA Hispanic patients used during the validation process. (PDF 568 kb) [file 13075_2018_1741_MOESM2_ESM.pdf]

Figure S1. Rheumatoid Arthritis Empowerment Scale for Hispanic patients (RAEH)

Validación de una escala para medición de empoderamiento en pacientes con artritis reumatoide del INCMNSZ.

Escala de empoderamiento de artritis reumatoide

Estimado paciente, le pedimos opine sobre los siguientes enunciados, al leerlos siempre ponga antes la frase "En general yo creo que", luego elija de entre las opciones de la derecha, la que mejor refleje su acuerdo o desacuerdo y marque una sola respuesta por frase anotando una cruz en la casilla. Ejemplo: ☒

En general, yo creo que…	Muy de acuerdo 5	De acuerdo 4	Ni de acuerdo ni en desacuerdo 3	En desacuerdo 2	Muy en desacuerdo 1
1. Puedo afrontar de manera positiva el estrés que me causa la artritis reumatoide.	☐	☐	☐	☐	☐
2. Puedo encontrar apoyo para cuidar de mi artritis reumatoide.	☐	☐	☐	☐	☐
3. Reconozco lo que me ayuda a estar motivado para cuidar de mi artritis reumatoide.	☐	☐	☐	☐	☐
4. Me conozco lo suficiente para elegir lo que más me conviene para el cuidado de mi artritis reumatoide.	☐	☐	☐	☐	☐
5. Conozco bien con que aspectos del cuidado de mi artritis reumatoide estoy insatisfecho.	☐	☐	☐	☐	☐
6. Soy capaz de alcanzar las metas que me he propuesto para mi artritis reumatoide mediante acciones concretas.	☐	☐	☐	☐	☐
7. Puedo hacer diferentes cosas para superar los obstáculos y lograr las metas que me he propuesto para mi artritis reumatoide.	☐	☐	☐	☐	☐
8. Puedo encontrar maneras para sentirme bien teniendo artritis reumatoide.	☐	☐	☐	☐	☐
