# Supplementary material for: Psychometric validation of an empowerment scale for Spanish-speaking patients with rheumatoid arthritis
Source: Arthritis Res Ther. 2018 Oct 30;20:244. doi: 10.1186/s13075-018-1741-6 (PMC6235224; doi:10.1186/s13075-018-1741-6)
Supplement: Supplementary file 3 — Table S2. Comparison of SWE-RES-23, S-HES and RAEH psychometric validation properties. Table shows the statistics of the tests carried out in the validation of the SWE-RES-23, S-HES, and RAEH scales. (PDF 301 kb) [file 13075_2018_1741_MOESM3_ESM.pdf]

Table S2. Comparison of SWE-RES-23, S-HES and RAEH psychometric validation properties.

	**SWE-RES-23**	**S-HES**	**RAEH**
Number of subscales/Number of items	5/23	8/8	8/8
(Mean±SD) score for the sample[1]	3.5±0.57	3.5±0.73	4.2±0.57
Floor and ceiling effect, %	2.2 and 15.1	<20 and <20	0.5 and 7.5
Cronbach α	0.920	0.890	0.862
ICC (95%CI), p value	NA	0.92 (NA), ≤0.001	0.79 (0.62-0., ≤0.001
% of the explained variance	64.1	52.4	52
Kaiser-Meyer-Olkin sampling (KMO)	0.895	0.890	0.884
Bartlett´s test of sphericity, p value	NA, p≤0.001	X^2=5425.72, p≤0.001	X^2=610.93, p≤0.001

IQ=Interquartile range; NA=not available. [1]Data available for the S-HES.
